# Production and Characterization of Polyethylene Terephthalate Nanoparticles

**DOI:** 10.3390/polym13213745

**Published:** 2021-10-29

**Authors:** Francesca Lionetto, Carola Esposito Corcione, Aurora Rizzo, Alfonso Maffezzoli

**Affiliations:** 1 Department of Engineering for Innovation, University of Salento, Via Arnesano, 73100 Lecce, Italy; carola.corcione@unisalento.it (C.E.C.); alfonso.maffezzoli@unisalento.it (A.M.); 2 CNR NANOTEC—Istituto di Nanotecnologia, c/o Campus Ecotekne, Via Monteroni, 73100 Lecce, Italy; aurora.rizzo@nanotec.cnr.it

**Keywords:** poly (ethylene terephthalate) nanoplastics, model nanoplastics, microplastics, annealing, DSC, marine plastics, ocean pollution, ball milling, nanoplastic formation

## Abstract

Microplastic (MP) pollution represents one of the biggest environmental problems that is further exacerbated by the continuous degradation in the marine environment of MPs to nanoplastics (NPs). The most diffuse plastics in oceans are commodity polymers, mainly thermoplastics widely used for packaging, such as polyethylene terephthalate (PET). However, the huge interest in the chemical vector role of micro/nanoplastics, their fate and negative effects on the environment and human health is still under discussion and the research is still sparse due also to the difficulties of sampling MPs and NPs from the environment or producing NPs in laboratory. Moreover, the research on MPs and NPs pollution relies on the availability of engineered nanoparticles similar to those present in the marine environment for toxicological, transport and adsorption studies in biological tissues as well as for wastewater remediation studies. This work aims to develop an easy, fast and scalable procedure for the production of representative model nanoplastics from PET pellets. The proposed method, based on a simple and economic milling process, has been optimized considering the peculiarities of the polymer. The results demonstrated the reliability of the method for preparing particle suspensions for aquatic microplastic research, with evident advantages compared to the present literature procedures, such as low cost, the absence of liquid nitrogen, the short production time, the high yield of the process, stability, reproducibility and polydisperse size distribution of the produced water dispersed nanometric PET.

## 1. Introduction

Nowadays, rapid population growth and the daily utilization of polymers for manufacturing non-reusable stuffs for various applications, such as packaging, cosmetics, textiles, detergents, greenhouse films, mulches, fishing nets, etc. cause large amounts of waste products and issues relating to their managing and disposal [1,2,3,4]. As a consequence, world production of municipal solid waste went from 1.3 billion tons in 1990 to 3.81 billion tons after 25 years [5], and plastics are a considerable percentage of this latter value [6,7]. In particular, in 2018, almost 29.1 million tons of plastic waste were collected in the European Union, of which 42.6% were incinerated with energy recovery through energy-from-waste, 32.5% were recycled and 24.9% were landfilled [8]. In addition, the waste management issues due to plastics can be ascribed not only to their excessive use but also to their small service life and slow degradation time [9]. Between 4.8 and 12.7 million tons of plastic waste are dispersed in the ocean each year [10]. Most of them derive from land-based activities, but a growing percentage arises from fishery activities waste [11]. Within Europe, around 85% of marine litter found on beaches is plastic. Around 43% of this marine litter is single use plastic, and 27% is fishing gear [12].

Within the polymer family, the highest impact is made by thermoplastic polymers such as polystyrene (PS), polyethylene (PE), polyvinyl chloride (PVC), polyurethane (PU), polypropylene (PP), and polyethylene terephthalate (PET) [13,14]. PET, a semicrystalline, lightweight, and water-resistant thermoplastic polymer, can be considered the largest used polymer in the production of packaging films, textiles and bottles, with a worldwide production of at least 30 million tons per year [15]. In addition, PET litter in the marine environment has lately been identified as one of the most dangerous types of waste for the environment, producing great damage, such as the entanglement of the marine species within plastic, or their ingestion of it [16,17]. Though PET could be recycled by using well known methods [18,19] several PET disposable items are discarded, accumulating in the environment. This litter undergoes dangerous photochemical, thermal and mechanical degradation, with the consequent production of fragments of PET of different size and morphologies [20]. Several papers report the presence of fragmented polymers, named ‘microplastics’, in drinking water [21,22], beverages [23] food [24] and the natural environment [25]. Microplastics (MPs) are polymer particles with a size between 5 mm and 1 µm [26], while larger particles, such as pellets, are named mesoplastics [27]. Moreover, the degradation of marine MPs due to protracted external light exposure, mechanical abrasion, and biodegradation can produce nanoplastics (NPs) with sizes lower than 1 μm [28,29,30]. Several authors reported that marine MPs are largely diffused in coastal environments and that they can be easily eaten by marine species [31]. Moreover, both microplastics (MPs) and nanoplastics (NPs) can adsorb and deliver chemicals present in marine environments and are derived from sea-based activities such as shipping, mariculture, offshore activities, etc., and land-based activities such as urban wastewater discharge, industrial and agricultural activities, mining, runoff from coastal areas, etc. [32]. Therefore, MPs and NPs can be considered one of the growing environmental contaminants with a significant impact on organisms [1,33]. MPs and NPs have also been detected in urban atmospheres as well as in remote and pristine environments, showing that atmospheric transport of MPs/NPs is also very significant [34,35].

Fundamental studies are required to understand the behaviour, fate and transport of ΜPs and NPs using representative model NPs. However, comprehensive biological and wastewater remediation studies relating to the effects of PET polymer nanoparticles on live organisms are still missing. Several studies have been performed on simplified NPs based on monodisperse polystyrene (PS) nanoparticles [36]. The commercial PS NPs are often obtained via water-dispersed processes with surfactants and preservatives, as reported by El Hadri et al. [36]. These PS NPs are often of a spherical shape and behave differently compared to irregularly shaped MPs found in environmental samples [37]. However, information on the importance of particle size, different surface chemistry and controlled particle degradation is still missing. The environmental relevance of micro/nanoplastics used in laboratories for a better understanding of in vivo behaviour and toxicological studies is a serious issue in the scientific community. In particular, in the case of PET NPs, the problem of PET contamination and the relationship between the migration/dispersion of PET NPs from one compartment to another and all the environmental compartments (terrestrial, aquatic, and atmospheric) has not been completely examined due the lack of easy and reproducible PET NPs production methods.

Currently, only a few methods have been reported for the production of PET nanoparticles, such as that proposed by Magrì et al. [38], who used laser ablation of polymer films in water, which produced PET nano-particles with a controlled size distribution. However, laser ablation is a very expensive process that requires expensive equipment and produces a very low yield. Another route for producing PET nanoparticles is based on the precipitation of NPs from a polymer solution of PET dissolved in a solvent, as proposed by Rodríguez-Hernández et al. [39] on fragments of PET drink bottles dissolved in a concentrated trifluoroacetic acid solution, or by Johnson et al. [13] on PET fibers dissolved in hexafluoroisopropanol. These methods allow the production of a large amount of PET micro/nanoparticles, without any chemical alteration, demonstrating their suitability for in vitro and in vivo studies to assess the potential impact of nanosized plastics in the environment. However, the methods require the use of toxic solvents, which are harmful for the operators during production, and require an additional procedure for their removal from the obtained NPs.

Another route to significantly reduce polymer particle size is based on mechanical milling, as studied by the authors at the University of Salento, where microplastics of different sizes were produced from PET industrial pellets by mechanical milling. Their suitability for toxicological studies on marine organisms has been assessed by Terlizzi and coworkers [40,41]. Mechanical milling, often combined with liquid nitrogen, has been very recently reported in the literature for the production of polymer micro- and nanoparticles, mostly made of polyethylene and polystyrene [36,37,42,43,44]. To the authors’ knowledge, only the works of Ji et al. [45] and Caldwell et al. [46] have applied mechanical milling to the production of PET NPs. Despite the simplicity of operation and the absence of toxic solvents, mechanical milling shows some limitations related to the low yield and long times required to obtain the nanometric sizes [45]. The long-lasting mechanical treatment could lead to polymer overheating and, consequently, to friction-induced thermal degradation or polymer agglomeration [42,46].

In order to increase the yield of nanometric fraction and to reduce the residence time in the mill, it is necessary to tailor the milling process in conjunction with the nature of the polymer. This issue has been neglected by recent studies on the topic, where trial and error approaches have led to long-lasting protocols of several milling hours, often with the use of liquid nitrogen, which is necessary to avoid polymer overheating. A ball mill is commonly used for brittle materials, typically ceramic materials [47,48], but, due to the viscoelastic behaviour of polymers, it is not easy to produce micrometric and nanometric polymer powders with this comminution technique. The fracture mechanisms during milling are, in fact, generally based on impact and shear. In the case of polymers, the brittle-ductile transition is, instead, crucial for the success of polymer milling to the nanometric size. In the authors’ opinion, this crucial aspect that has not yet been investigated in the previous literature on nanoplastic production finds confirmation in the experimental observations of Kuhn et al. [44], where different polymer types, such as those found in beached litter, cannot be successfully milled together and need separation.

The present work, therefore, aims at studying an important issue, until now not considered in the literature, on the production of polymer micro- and nanoplastics by the milling process. It is focused on the relationship between the PET semi-crystalline structure and mechanical treatment, by investigating the structural changes induced by milling, and using calorimetric and structural techniques. The aim of this work is the development of a fast and reliable mechanical method for the laboratory production of PET nanoparticles at ambient temperature, accounting for the structural changes in the semicrystalline polymer during milling. This has been accomplished by a deep investigation of the evolution of crystallinity by calorimetric and X-Ray diffractometric analysis. The understanding of the structural changes during milling has been crucial for the correct choice of the processing conditions useful for achieving nanometric PET dispersions in water by minimizing the residence time in the mill. For this purpose, starting from DSC and XRD analysis of PET pellet and powders milled for different times, several multi-step reduction size processes have been developed, monitoring the size and the distribution of the treated PET particles by laser diffraction.

## 2. Experimental

### 2.1. Preparation of the Aqueous Dispersion of PET Nanoparticles

The investigated material was RT52 polyethylene terephthalate (Invista Resins & Fibers GmbH, Gersthofen, Germany) in the form of a pellet with 4 mm average length and an intrinsic viscosity of 0.634–0.638 dL/g.

**Figure 1:**
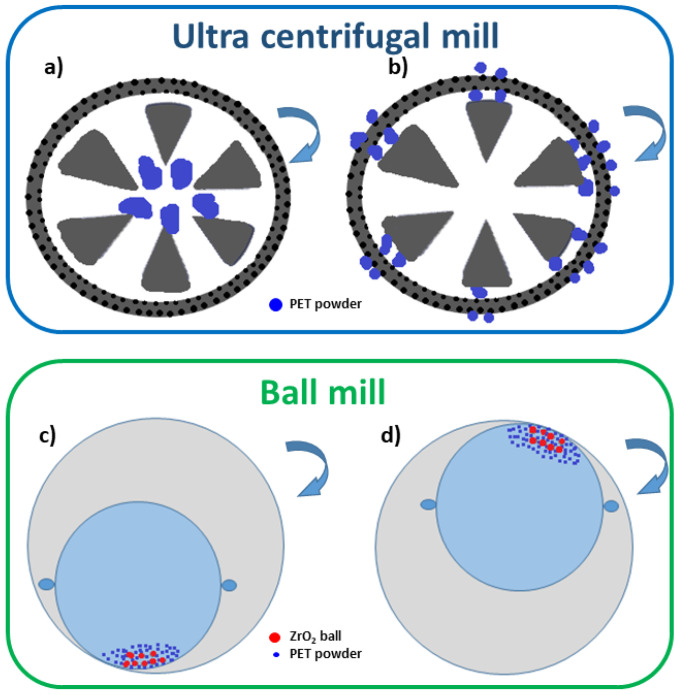
Schematic representation of the milling process in (**a**,**b**) an ultra-centrifugal mill and (**c**,**d**) a ball mil (The powder particles are blue, while the milling balls are red).

In order to reduce the pellet to micrometric powder, PET pellets were firstly dry milled using a RETSCH ZM100 Ultra Centrifugal mill (RETSCH GmbH & Co., Düsseldorf, Germany), at 14,000 rpm by adopting three milling consequent cycles with different sieves. In particular, 500 μm, 250 μm and 80 μm mesh sieves were used, labeled as S500, S250, S80, respectively. A schematic representation of the grinding process in the ultra-centrifugal mill is reported in Figure 1a,b. The centrifugal acceleration projected the particle out from the grinding chamber with great energy, leading to pre-crushing by impact on the wedge-shaped rotor teeth moving at 14,000 rpm (Figure 1a). The polymer particles were then finely ground by the shearing action between the rotor and the ring sieve (Figure 1b).

After ultra-centrifugal milling, a further milling stage in a ball mill was necessary to reduce the micrometric powders to nanometer scale. A ball mill is a simple and cost-effective way of producing homogeneous and ultrafine powders, mostly ceramic and metallic powders [49,50]. The powders were ball milled in a zirconia (ZrO_2_) jar (0.3 L), using zirconia balls in an ambient atmosphere; the mechanical milling was performed in a ball mill, model S/1 1000B (Ceramic Instruments S.r.l, Sassuolo Modena, Italy), operating at 390 rpm. The particles were finely reduced to nanometric scale thanks to the high-energy collisions with the zirconia balls. Friction resulted from the powder and the balls, alternately rolling on the inner wall of the bowl and striking the opposite wall (Figure 1c,d). The operating conditions of the ball mill has been optimized as will be described in the result section.

Three ball milling procedures, called A, B and C, have been developed by changing process parameters. The experimental details will be reported in the Results section since they have been obtained by an optimization of the research activity.

### 2.2. Characterization of the Micrometric and Nanometric Particles

The structural changes of the PET powders during milling were analysed by differential scanning calorimetry (DSC) and X-Ray diffraction (XRD). DSC analysis was performed on a Mettler Toledo 822 (Mettler Toledo, Greifensee, Switzerland) instrument under a nitrogen flux of 60 mL/min, applying a heating scan between 25 and 300 °C at 10 °C/min. Each measurment was the average of at least three replicates.

XRD analysis was carried out using an X-Ray diffractometer (Rigaku, Tokyo, Japan) with a CuK*α* radiation (*λ* = 1.5418 A°) in the step scanning mode recorded in the 2*θ* range of 10°–60° with a step size of 0.02° and step duration of 0.5 s. Three replicates for each measurment were performed.

The analysis of particle size distribution was performed by laser diffraction using a CILAS 1190 multi-laser particle size analyzer and Particle Expert^®^ software (CPS Us, Inc., Madison, WI, USA). At least three measurements were performed for each dispersion typology. The Mie theories [51] were applied for the particle size measurement and the reflective index of the material was defined in Particle Expert software.

Scanning electron microscopy (SEM) was performed on samples prepared by spin coating a suspension of nanoplastics on conductive indium thin oxide (ITO) coated glass substrates. The morphological SEM characterization was carried out by a ZEISS Sigma 300 field emission SEM (FE-SEM) instrument in high vacuum and high-resolution mode, equipped with a Gemini column by SE detector. A 5 kV voltage acceleration was used. The statistical analysis of nanoplastic sizes was performed by measuring the particle diameters by the ImageJ program on more than 200 nanoparticles for each population.

## 3. Results

### 3.1. Analysis of the Structural Changes during Dry Milling in an Ultra Centrifugal Mill

**Figure 2:**
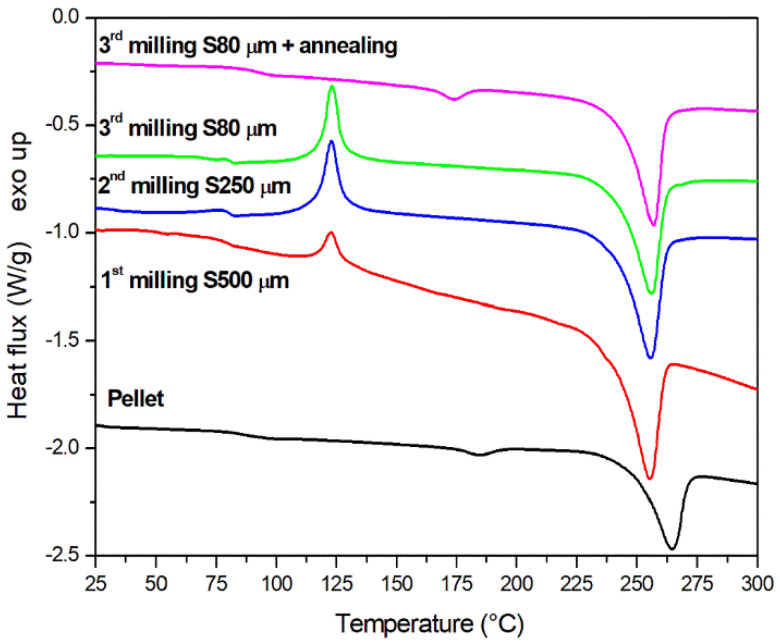
DSC dynamic scans of PET pellet (before milling) and powders after consecutive milling steps in an ultra centrifugal mill with different mesh sieves. Annealing was performed at 160 °C.

The combined action of impact and shear during milling is expected to result in a very fast temperature increase of the polymer particle, which, consequently, should greatly affect its structure and the efficacy of the milling process. Thermal analysis has then been used to investigate the structural changes induced by milling in PET samples and to evaluate a possible route to mitigate these changes, such as, for example, an annealing treatment. The DSC dynamic scans of PET pellets (before milling) and powders after consecutive milling steps in an ultra-centrifugal mill with different mesh sieves are reported in Figure 2.

The DSC scan of semi-crystalline pellets before milling, reported in Figure 2, presents a melting peak at 263 °C, expected for polyethylene terephthalate, and a small melting peak at 187 °C due to the presence of a homogenous amount of lamellae of similar thickness, as discussed below. The corresponding latent heats are 33.8 J/g and 2.7 J/g, respectively.

From the calorimetric scans, the degree of melting *X_DSC_* can be determined from the following equation:

(1) $$X_{DSC}{}_{}(T)=\frac{\Delta H(T)}{\Delta H_{mTOT}}$$

where Δ*H(T)* is the enthalpy absorbed from the beginning of melting to the temperature T, calculated as an integral of the DSC signal after subtraction of the baseline [52]. Δ*H_mTOT_* has been assumed to be the total heat absorbed in the melting process by a fully crystalline polymer, equal to 140.1 J/g [53,54]. The average degree of crystallinity of PET pellets, obtained by DSC measurements, is equal to 0.26.

The effect of consecutive milling steps in an ultra centrifugal mill on the melting behavior of PET can be inferred from Figure 2. The thermograms of milled powders present both a depletion in the glass transition temperature (*T_g_*) and a crystallization peak whose area (Δ*H_c_*) increases with the consecutive milling steps at different mesh sieves, as reported in Table 1. This leads to a progressive reduction of the degree of crystallinity, as calculated from the DSC enthalpy areas. In the presence of a cold crystallization enthalpy, the degree of crystallinity has been calculated by subtracting the heat of cold crystallization (Δ*H_c_*) from the heat of melting (Δ*H_m2_*) and dividing by the heat of fusion for a fully crystalline PET. This procedure could help to remove the contribution of the crystallization occurring during the DSC scan, immediately above *T_g_*. However, it should be underlined that the thermal treatment during the DSC measurement can lead to wrong absolute values of the crystallinity of PET. Therefore, the degree of crystallinity calculated from DSC can be named as an “apparent” crystallinity [55]. The calorimetric results presented in Figure 2 and Table 1 demonstrates that the semicrystalline PET shows a tendency to become amorphous during mechanical grinding. The observed phenomenon can be attributed to melting followed by quenching during comminution in the ultra-centrifugal mill, which is likely responsible for an increase of the amorphous fraction of the milled powder. A similar behavior has been reported during solid state shear milling of polypropylene [56] and during ball milling of polyethylene terephthalate [55].

The amorphization induced by milling is associated with an increase in the sample toughness and a reduction of the polymer brittle-ductile transition. This could lead to the absence of fracture during grinding and to polymer welding as a consequence of high local temperature increases.

In order to alleviate this phenomenon, a possible strategy could be to increase the polymer crystallinity after each grinding step. To this aim, an annealing treatment has been applied on the PET powders to erase the amorphous fraction induced by milling. It is known that the annealing of a semi-crystalline polymer at a high temperature, close but lower than the melting temperature, leads to a thickening of crystalline lamellae with a consequent increasing of the degree of crystallinity. Generally, an annealing temperature in the range 190–220 °C is reported in the literature for polyethylene terephthalate [54]. However, in the analyzed pellets, the presence of a prevalent amount of crystal lamellae that melt at 187 °C with an onset temperature above 173 °C, has driven the choice of a lower annealing temperature than that reported in the literature. In this study, the annealing treatment has been carried out at 160 °C for 4 h followed by a slow cooling to room temperature.

**Figure 3:**
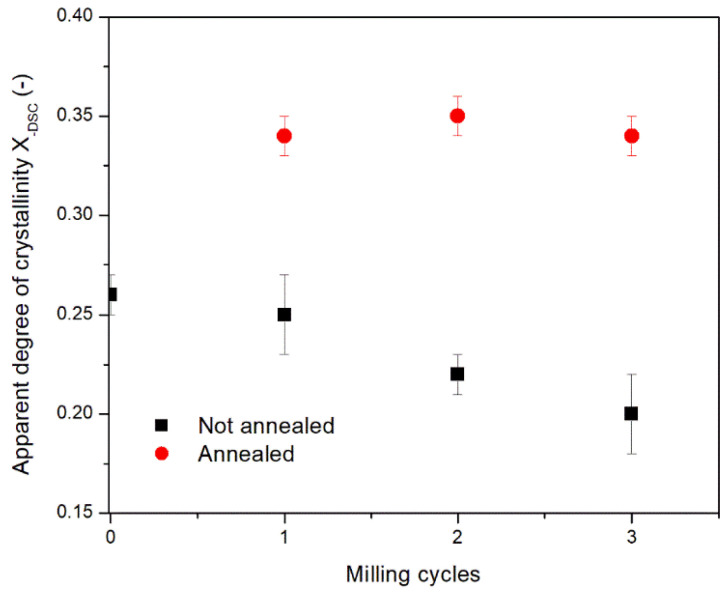
Effect of the number of milling cycles on the apparent crystallinity of PET samples measured by DSC before and after annealing at 160 °C.

After annealing, the powders show a lower amorphous content, as proved by the lack of the crystallization peak in all the DSC dynamic scans. For sake of clarity, in Figure 2 only the thermogram relative to the powder milled with S80 sieve and annealed (magenta curve) has been reported. The DSC scans obtained on the other powders after annealing present a similar behavior with a very close degree of crystallinity, obtained from the enthalpy area. As reported in Figure 3, the apparent crystallinities of the annealed powders are very close to each other regardless the number of milling steps. This result confirms that the structural changes induced by milling are reversible and can be erased by a proper annealing treatment. The small peak appearing at about 10 °C above the annealing temperature indicates that a homogeneous population of lamellae resulted from annealing (see Table 1). After the onset of melting of these lamellae at a temperature of about 10 °C above the annealing temperature, the molten polymer can again crystallize being above the glass transition temperature (*T_g_*) and below the melting temperature (*T_m_*) in an exothermic process. After the onset of melting of these lamellae, melting and recrystallization occurs simultaneously, giving a zero net DSC signal, resulting on the concurrence of equivalent endothermic and exothermic processes [57,58]. The crystals formed above 170 °C during heating are then characterized by an onset of melting at about 240 °C.

Compared to the *T_g_* of the starting pellet (84 °C) and the milled powders (79–81 °C), the annealed powders also present an increased *T_g_*, in the range 91–93 °C. This is likely due to the restrictions imposed to the molecular chains by the increased crystalline fractions. The increased *T_g_* is observed in a polymer when its crystallinity or degree of crosslinking increases [59]. Therefore, the observed increase in *T_g_* and crystallinity is expected to increase the polymer brittleness, which is a crucial parameter for an effective milling.

**Figure 4:**
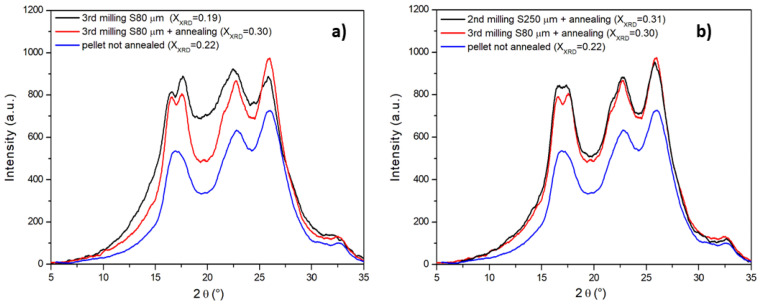
(**a**) Effect of annealing on the XRD spectra of powders milled with the S80 sieve; (**b**) XRD spectra of annealed powders milled with different sieves.

XRD analysis has confirmed the ability of annealing to bring crystallinity at its maximum value as shown in Figure 4a, where the spectra of annealed and not annealed powders milled with a S80 μm sieve are compared with the spectrum of the original untreated PET pellet.

The XRD spectra show crystalline peaks at 2θ = 16.5°, 17.6°, 22.5°, 25.9°, corresponding to the (0 $\bar{1}$ 1), (0 1 0), ($\bar{1}$ 1 0) and (1 0 0) planes, respectively (XRD JCPDS No. 50-2275) (Table S1, Supplementary material) [60]. The degree of crystallinity (*X_XRD_*) has been calculated from the XRD pattern using the integrated areas under the crystalline peaks *A_c_* and broad amorphous halo *A_a_*:

(2) $$X_{XRD}=\frac{A_{c}}{A_{c}+A_{a}}$$

The averaged degree of crystallinity *X_XRD_* are reported in Table 2.

**Figure 5:**
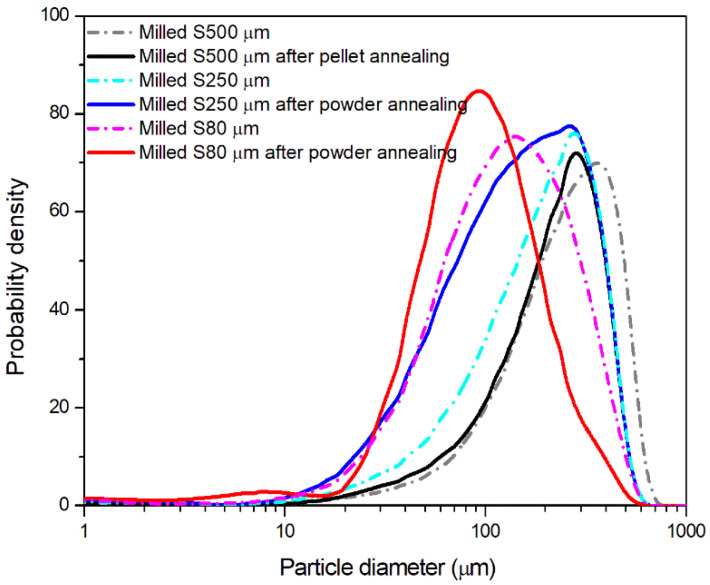
Size distribution of the powders obtained from ultra-centrifugal milling.

The increased crystallinity obtained after annealing lead to a more efficient and homogenous comminution in the ultra-centrifugal mill, as demonstrated by the size distribution measured by laser diffractometry reported in Figure 5, where the density distribution represents the probability of finding a particle with a diameter D in the population. The powders milled after annealing present, in fact, a distribution with a peak centered at lower diameters or with a higher content at lower sizes.

The laser diffraction results suggest the efficiency of the annealing stage in increasing PET crystallinity. This should also affect the transition between ductile fracture and brittle failure, as known from the literature [61,62]. Therefore, it can be concluded that the choice of proper annealing parameters can contribute to improve the size-reducing process of PET powders by promoting the transition from ductile to brittle behavior especially at high deformation rates such as that reached during the grinding process.

### 3.2. Optimization of Wet Ball Milling for Producing PET Nanoplastics

Starting from the previous experimental results, the second part of the work has been focused on the optimization of the wet milling conditions in a ball mill in order to set-up a procedure able to reduce the process time for obtaining PET nanoparticles with a polydisperse distribution and high yield. For this purpose, three multi-step milling procedures have been performed in a ball mill, monitoring the size and the distribution of the treated PET particles by a particle size analyzer. Particular attention has been devoted both to the choice of the milling and pause times and the initial structure of the starting polymer powder. In ball milling of polymers, grinding is also strongly dependent on the mechanical properties of materials, which can prevent the formation of the desired granulometry. The proper choice of milling/pause time can keep the polymer temperature under control, avoiding a local overheating due to the high number of impacts. Moreover, the viscoelastic properties of the polymer can affect the efficiency of the milling process, eventually preventing the fracture and leading to polymer agglomeration by welding during milling.

**Figure 6:**
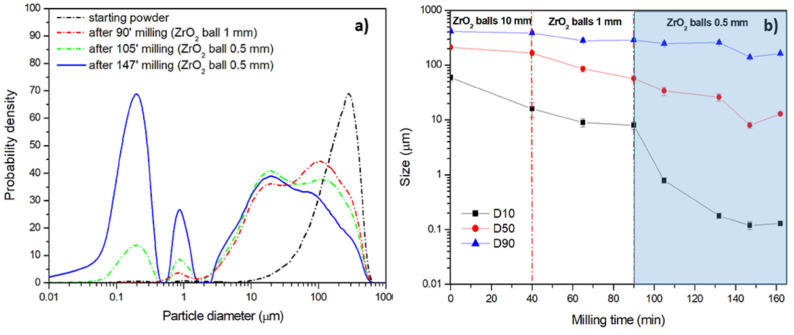
Procedure A: (**a**) particle size distribution at different milling times and ball diameters and (**b**) effect of milling time on average size (at 10%, 50% and 90% by volume of the analyzed particles).

The first procedure, named Procedure A, involves milling steps carried out with ZrO_2_ spheres of homogeneous diameter, which is reduced in three consecutive milling cycles characterized by different milling steps, as reported in Table 3. As an example, using 10 mm balls, the powders were subjected to milling for 5 min and pause for 5 min. With 1 mm and 0.5 mm balls, the milling time was 3 min and pause time was 7 min. The pause time was necessary to keep low temperature inside the jar. The size distribution of the aqueous dispersions at different milling times are compared with the starting powders in Figure 6a, using an average initial D50 size equal to 212 μm, which indicates that 50% by volume of the analyzed particles are below 212 μm. The milling with smallest balls (0.5 mm) seems more effective in achieving the nanometric scale. A trimodal pattern with a wide size distribution in the range 2.5–600 μm, a micrometric distribution centered at about 1 μm and a significant nanometric fraction center at about 300 nm, was obtained.

The reduction of particle size has been monitored at regular time intervals. Figure 6b shows the temporal evolution of the D10, D50, and D90 values which, respectively, indicate that 10%, 50% and 90% by volume of the analyzed particles are below the corresponding values. It appears evident that the milling steps with 10 mm ZrO_2_ balls are ineffective in the size reduction, except for D10 values, and can be removed, enabling the saving of both milling time and post-milling time, which are necessary for sieving and changing the milling ball. This result disagrees with a recent work [36] on the production of HDPE and PES nanoparticles, where balls with diameters lower than 5 mm are not recommended due to agglomeration problems. These problems are most likely related to the temperature increase during milling, which can lead to ineffective impacts with possible welding of powders rather than the fracture. In the present case, the choice of a proper pause time enables the reduction of the local temperature increase caused by ball collisions. The temperature of the dispersion inside the jar has been measured with a K-type thermocouple after one milling and one pause step. Values lower than 35 °C have been measured, which are much lower than the glass transition temperature of the polymer. The milling steps with 1 mm ZrO_2_ balls present small but significant size reductions. The highest size reduction, especially for D10 values, are achieved using 0.5 mm ZrO_2_ balls.

The time of this procedure has been established as the minimum time necessary to obtain the maximum size reduction, after which two consecutive measurements give constant size values or, eventually, slight particle agglomeration. In Figure 6b it can be observed that further milling steps for times longer than 147 min, i.e., 162 min in Figure 6b, leads to a slight particle agglomeration. Therefore, 147 min has been considered as the minimum time required to achieve the nanometric dispersions under this process conditions.

Procedure A leads to a homogenous nanometric dispersion in a time span of 147 min of ball milling, with an overall process time of 351 min, also considering the pause time. The yield of the nanometric fraction below 1 μm has been calculated from the cumulative curve obtained from laser diffractometry, which represents the percentage of particles which are smaller than a diameter *d* and is a simple integral of the distribution curve. As reported in Table 3, a yield of the nanometric fraction lower than 1 μm equal to 33.4% by volume has been obtained using Procedure A.

**Figure 7:**
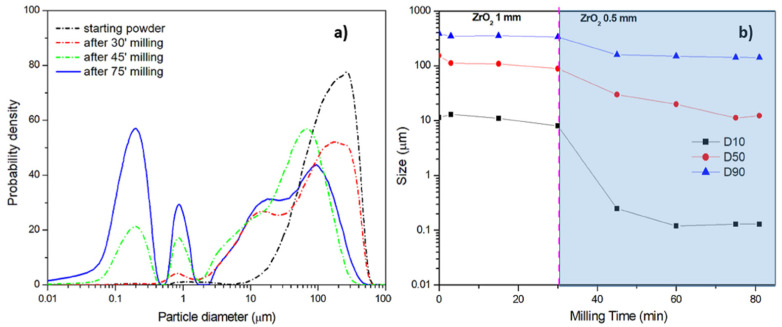
Procedure B: (**a**) size distribution at different milling times and ball diameters and (**b**) effect of milling time on average size (at 10%, 50% and 90% by volume of the analyzed particles).

The second procedure, named Procedure B, has been performed by using only two types of ZrO_2_ balls, with diameters equal to 1 mm and 0.5 mm, and microparticles with an average D50 size equal to 190 μm, which have been annealed before ball milling. The aqueous dispersion was ball milled for 3 min, followed by a pause time of 6 min. As previously demonstrated, the annealing treatment should provide high crystallinity and mechanical properties, thus leading to effective impacts to reduce the particle size. The size distribution of the aqueous dispersions reported in Figure 7a at different milling times shows that in this case a trimodal distribution was also achieved. Figure 7b does not a show substantial decrease in the particle size when 1 mm ZrO_2_ balls were used., Therefore, this ball size was not considered for Procedure C. However, the great reduction of particle size provided by 0.5 mm ZrO_2_ balls is evident, especially for the D50 and D10 values.

**Figure 8:**
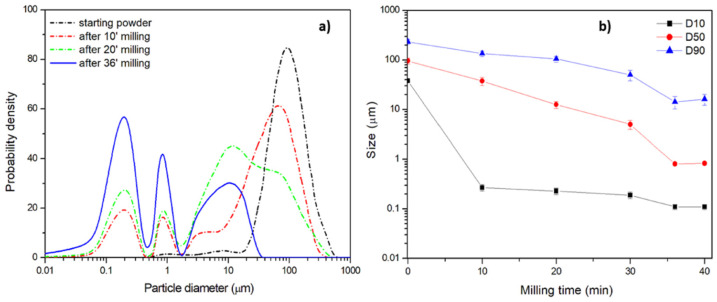
Procedure C: (**a**) size distribution at different milling times and (**b**) the effect of milling time on average size (at 10%, 50% and 90% by volume of the analyzed particles).

The last procedure, named Procedure C, involves the use of only one type of ZrO_2_ ball, with 0.5 mm diameter, and smaller starting powders with an average D50 size equal to 120 μm, annealed before ball milling. Moreover, the high number of balls implicates a high number of impacts and the possible risk of an overheating in the jar. For this reason, the milling time has been reduced to 2 min, followed by 5 min of pause. A trimodal distribution appears quickly after 10 min of milling and the nanometric content increases at the expense of the micrometric one (Figure 8a). In Figure 8b the average size as a function of milling time is reported. The size rapidly decreases, reaching a plateau after 36 min of milling which corresponds to an overall process time of 120 min including the pause time. This indicates that the choice of the milling and pause parameters have been appropriate to avoid particle agglomeration during milling.

The obtained significant reduction of the process time can be inferred from Table 3. Even if the nanometric sizes achieved by the three procedures are comparable, Procedure C is more efficient, with the highest yield of nanometric fraction below 1 μm, equal to 36.4% by volume, achieved in the lowest time. The milling time of about 36 min is very low if compared to the 3 h reported for PET nanoplastics with a different ball mill but with the same ZrO_2_ ball diameter [46]. The effectiveness of ball milling with hard small spheres can be considered a process very close to what can occur on sea shores, where hard sand granules hit meso- and microplastics at low rates but for a time incommensurably longer.

**Figure 9:**
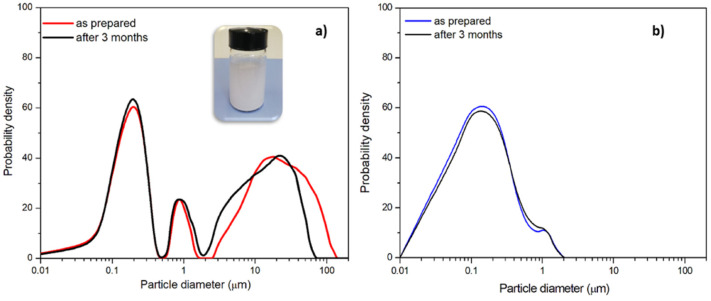
Dispersion stability over time: (**a**) aqueous dispersion of nanometric PET prepared according to procedure C; (**b**) aqueous dispersions of nanometric PET sieved before ball milling and then prepared according to procedure C.

Depending on the application of the nanoplastics, the micrometric annealed powders can also be sieved before the ball milling process to eliminate the higher size fraction and achieve a bimodal nanometric distribution, as reported, for example in Figure 9b. A very good stability over time has been proved by measuring the particle size distribution after several months from preparation of the aqueous dispersion, without using any surfactant. A good overlapping of the curves can be observed in Figure 9. This procedure allows for the preparation of polymer nanoparticles without surfactants, by analogy with the long wearing/weathering process occurring in the sea. For this reason, the stability of nanoparticle dispersion also occurs in the sea, making them more easily eaten by marine organisms despite that they have a density higher than that of sea water.

**Figure 10:**
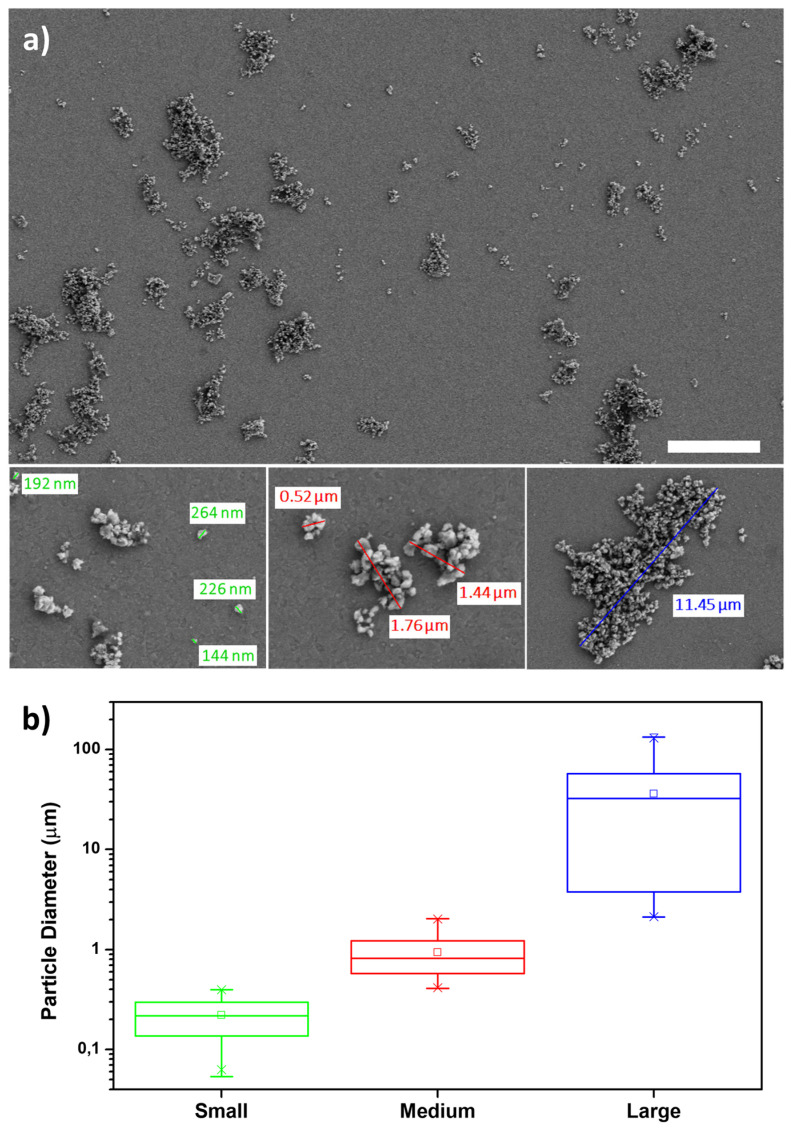
(**a**) Representative SEM images of the sample obtained according to procedure C (scale bar is 10 μm). A zoom on the three different populations founded is also reported. (**b**) Statistical analysis of the micro/nanoplastics size distribution observed by SEM imaging.

The scanning electron microscopy (SEM) inspection shows samples composed of small particles with sizes variable in the range of 70–400 nm, which aggregate forming bigger clusters (Figure 10a). Three populations of clusters have been detected, namely small, formed by 1 or 2 particles, medium, and large. The medium size clusters range from 400 nm to 2 μm; the large size clusters are above 2 μm and can reach a diameter from tens to hundreds of microns (Figure 10b).

## 4. Conclusions

**Figure 11:**
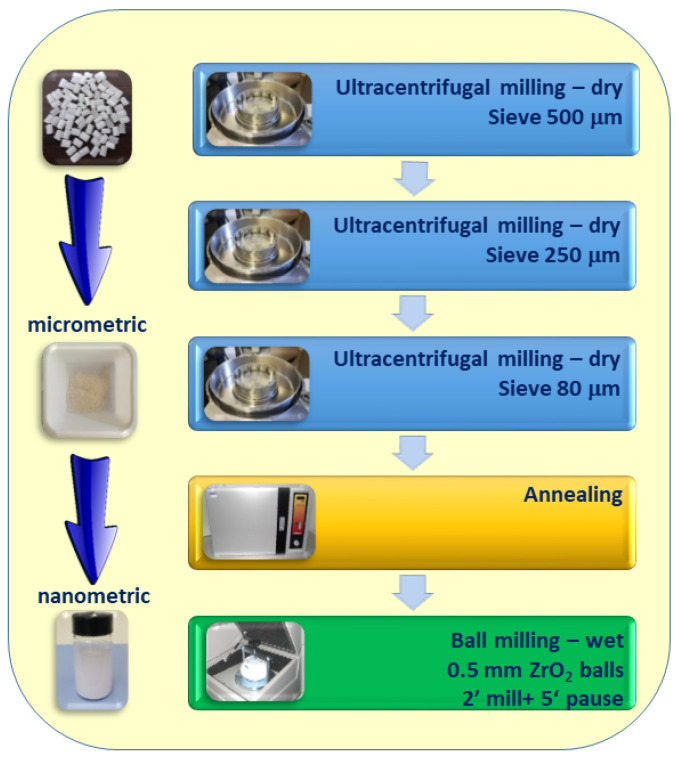
Schematic representation of the optimized Procedure C.

In this study, a simple and effective size reduction process was developed to prepare PET nanoplastics from pellets, as illustrated in Figure 11. The understanding of the structural changes of PET during milling has been crucial for the correct choice of the processing conditions useful for achieving nanometric PET dispersions in water by minimizing the residence time in the mill. The optimized procedure requires three stages of very fast ultra-centrifugal dry milling with three different sieves (500 μm, 250 μm, 80 μm) in order to reduce the pellet size with an average length of 4 mm down to micrometric powders with an average size of 100–120 microns. Then, the powders are subjected to an annealing treatment at 160 °C for 4 h, followed by a slow cooling to room temperature. The annealed powders are wet ball milled with 0.5 mm ZrO_2_ balls until reaching the nanometric size. The most important advantage is the possibility of obtaining stable and reproducible water dispersed nanometric PET in a shorter time compared to the time reported in the literature required for milling thermoplastic polymers. Another advantage of the proposed method consists in achieving a polydisperse size distribution that makes easier separation by size for further studies of interaction with marine organisms. The observed size heterogeneity could be similar to that present in the real nanoplastics gathered in the marine environment [28,46], which are produced by the degradation of primary and secondary microplastics with a ball milling-like process where small hard sand granules play the role of zirconia balls in the proposed laboratory experiments. However, if for a specific experiment, a size fraction of NPs is required, the aqueous dispersion can be subject to centrifugation and filtration in order to collect, for example, only a close nanometric fraction.

A further advantage of the proposed method is the possibility to obtain nanoparticles starting from powders with different sizes and different origins, i.e., industrial pellets or recycled bottles. In addition, the proposed method is not expensive and does not need liquid nitrogen. Finally, the obtained dispersions are already present in water and could be potentially ready for successive target applications, such as sorption studies of contaminants in the marine environment, analyses of the nanoplastic interactions with biological systems, and research on the removal approaches of nanoplastics from the aquatic environment.

## Figures and Tables

**Table 1:** DSC results for PET pellet and powders after consecutive milling steps in an ultra-centrifugal mill with different mesh sieves.

	T_g_ (°C)	T_c_ (°C)	ΔH_c_ (J/g)	T_m-1_ (°C)	ΔH_m-1_ (J/g)	T_m-2_ (°C)	ΔH_m-2_ (J/g)
Pellet	84.0 ± 1.0	-	-	187.2 ± 5.1	2.7 ± 0.1	263.4 ± 0.3	33.8 ± 0.7
1st milling S 500 µm	79.8 ± 0.3	125.3 ± 3.0	9.6 ± 1.0	-	-	253.9 ± 1.1	44.8 ± 4.1
2nd milling S 250 µm	81.0 ± 0.6	123.0 ± 0.2	16.3 ± 2.9	-	-	255.5 ± 0.5	46.9 ± 3.5
3rd milling S 80 µm	80.6 ± 0.9	122.3 ± 0.4	20.9 ± 1.1	-	-	256.1 ± 0.3	48.5 ± 1.6
1st milling S 500 µm+ annealing	93.8 ± 0.4	-	-	171.5 ± 0.2	1.8 ± 0.2	255.6 ± 0.3	46.2 ± 1.1
2nd milling S 250 µm+ annealing	92.7 ± 0.3	-	-	173.9 ± 0.3	2.5 ± 0.3	256.6 ± 0.2	47.1 ± 0.3
3rd milling S 80 µm+ annealing	91.5 ± 0.3	-	-	173.4 ± 0.3	3.2 ± 0.3	256.3 ± 0.1	44.8 ± 0.2

**Table 2:** Degree of crystallinity obtained from XRD measurements.

	*X_XRD_* (-)
Pellet not annealed	0.22
S250 not annealed	0.20
S250 annealed	0.31
S80 not annealed	0.19
S80 annealed	0.30

**Table 3:** Experimental ball milling conditions and results for the different procedures.

Procedure	Starting Powder D_50_ (μm)	ZrO_2_ Ball (mm)	Milling Conditions *	Milling Time (min)	Overall Time (min)	D_10_ (µm)	D_50_ (µm)	D_90_ (µm)	Nanometric Yield ** (% by Volume)
A	212 ± 23	10	5 min (m)–5 min (p)	147	351	0.13 ± 0.01	8.60 ± 0.71	141.10 ± 1.41	33.4 ± 7.7
		1	3 min (m)–7 min (p)						
		0.5	3 min (m)–7 min (p)						
B	190 ± 21 annealed	1	3 min (m)–6 min (p)	75	252	0.13 ± 0.02	11.21 ± 0.65	142.64 ± 1.69	32.7 ± 5.5
		0.5	3 min (m)–6 min (p)						
C	120 ± 18 annealed	0.5	2 min (m) 5 min (p)	36	120	0.11 ± 0.01	0.81 ± 0.01	14.21 ± 1.56	36.4 ± 11.3

*: m = milling; p = pause, **: yield of the nanometric fraction with a size lower than 1 µm.

## Supplementary Materials

The following are available online at https://www.mdpi.com/article/10.3390/polym13213745/s1, Table S1: Extract from XRD JCPDS No. 50-2275.
