# Supplementary material for: Production and Characterization of Polyethylene Terephthalate Nanoparticles
Source: Polymers (Basel). 2021 Oct 29;13(21):3745. doi: 10.3390/polym13213745 (PMC8587476; doi:10.3390/polym13213745)
Supplement: Supplementary file 1 [file polymers-13-03745-s001.zip › polymers-1382018-supplementary.pdf]

Production and Characterization of Polyethylene Terephthalate Nanoparticles

Francesca Lionetto *, Carola Esposito Corcione and Alfonso Maffezzoli

Table S1. Extract from XRD JCPDS No. 50-2275.

2θ	Int	h	k	l
16.305	306	0	$\bar{1}$	1
17.745	370	0	1	0
21.463	218	$\bar{1}$	1	1
22.724	616	$\bar{1}$	1	0
24.120	131	0	1	1
24.966	135	$\bar{1}$	1	2
26.113	999	1	0	0
28.149	252	1	$\bar{1}$	1
